# Design Paper: The Short- and Long-term Effects of Inspiratory Muscle Training in Patients with Idiopathic Pulmonary Fibrosis—A Multicenter Randomized Controlled Trial (the Breathe IPF Trial)

**DOI:** 10.31662/jmaj.2026-0064

**Published:** 2026-08-21

**Authors:** Akihito Okada, Hiroaki Nakagawa, Tomohiro Handa, Yuta Kono, Naozumi Hashimoto, Yohei Oshima, Kohei Ikezoe, Yoshinari Nakatsuka, Naoya Tanabe, Susumu Sato, Kenichi Kobayashi, Ryota Kikuchi, Mayuko Ishiwari, Mariko Kogami, Shinji Abe, Yoshitsugu Inoue, Kohei Fujita, Akiko Nakano, Eiji Nakatani, Kenichi Yoshimura, Hirotsugu Ohkubo

**Affiliations:** 1 Department of Respiratory Medicine, Allergy and Clinical Immunology, Nagoya City University Graduate School of Medical Sciences, Nagoya, Japan; 2 Division of Respiratory Medicine, Department of Internal Medicine, Shiga University of Medical Science, Otsu, Japan; 3 Department of Advanced Medicine for Respiratory Failure, Kyoto University Graduate School of Medicine, Kyoto, Japan; 4 Department of Respiratory Medicine, Tokyo Medical University Hospital, Tokyo, Japan; 5 Department of Respiratory Medicine, Fujita Health University, Toyoake, Japan; 6 Rehabilitation Unit, Kyoto University Hospital, Kyoto, Japan; 7 Department of Respiratory Medicine, Nagoya City University East Medical Center, Nagoya, Japan; 8 Department of Respiratory Medicine and Allergy, Nagoya City University Midori Municipal Hospital, Nagoya, Japan; 9 Department of Biostatistics and Health Data Science, Graduate School of Medical Sciences, Nagoya City University, Nagoya, Japan

**Keywords:** idiopathic pulmonary fibrosis, pulmonary rehabilitation, inspiratory muscle training, quality of life

## Abstract

**Introduction:**

Pulmonary rehabilitation is beneficial for patients with idiopathic pulmonary fibrosis (IPF) but is often limited by poor adherence. Inspiratory muscle training (IMT) may represent a more feasible, home-based alternative. This trial aims to evaluate the short- (8-week) and long-term (56-week) effects of IMT using the POWERbreathe Medic Plus in patients with IPF.

**Methods:**

This multicenter, open-label, parallel-group, randomized controlled trial will assess the efficacy and safety of device-based IMT. Eligible participants (aged 40-85 years) with stable IPF and modified Medical Research Council dyspnea grades 1-3 will be randomly assigned (1:1) to an IMT group or a control group receiving usual care. The primary endpoint is the change in the St. George’s Respiratory Questionnaire activity domain score from baseline to week 8. Secondary endpoints include pulmonary function, exercise capacity, dyspnea, and quality of life over 56 weeks.

**Results:**

This trial will clarify the short- and long-term effects of IMT, as well as its safety, in patients with IPF.

**Conclusions:**

This study is the first to evaluate both the short- and long-term effects of IMT on quality of life in IPF.

**Protocol Registration:**

The Japan Registry of Clinical Trials (jRCT1042250003).

## Trial Background and Rationale

Idiopathic pulmonary fibrosis (IPF) is a chronic, progressive, and ultimately fatal interstitial lung disease characterized by irreversible lung fibrosis. Although antifibrotic agents such as nintedanib, pirfenidone, and nerandomilast can slow disease progression, they do not completely halt the fibrotic process ^(1), (2), (3)^. Chronic dyspnea and fatigue are among the most disabling symptoms of IPF and markedly impair patients’ health-related quality of life (HRQOL) ^(4)^. Accordingly, improving HRQOL, in addition to prolonging survival, has become a major therapeutic goal.

Dyspnea, the predominant symptom of IPF, often leads patients to restrict physical activity, resulting in deconditioning, muscle weakness, and reduced exercise capacity. This vicious cycle further compromises daily functioning and quality of life ^(5)^. Pulmonary rehabilitation can improve exercise tolerance and HRQOL in patients with IPF and other interstitial lung diseases ^(6)^. However, adherence to conventional programs—such as limb muscle strengthening or aerobic training—is frequently suboptimal because of disease progression, limited accessibility, and practical constraints ^(7)^. Therefore, feasible, home-based rehabilitation strategies that can be performed safely and consistently are needed.

Inspiratory muscle training (IMT) has emerged as a promising approach that directly targets inspiratory muscle dysfunction while requiring minimal physical exertion. IMT induces structural and functional adaptations of the diaphragm; ultrasonographic studies demonstrate increases in diaphragm thickness and maximal inspiratory pressure, indicating hypertrophy and enhanced contractile function ^(8), (9), (10)^. These physiological adaptations provide a plausible mechanistic basis for potential improvements in dyspnea and activity-related limitations.

In patients with chronic obstructive pulmonary disease, meta-analytic evidence indicates that respiratory muscle training improves 6-minute walk distance (6MWD), dyspnea, pulmonary function indices, respiratory muscle strength, and quality of life ^(11)^. Similarly, a randomized trial in adults recovering from COVID-19 demonstrated that 8 weeks of IMT produced clinically meaningful improvements in breathlessness, respiratory muscle strength, and functional capacity ^(12)^.

Evidence is also emerging in interstitial lung disease. A single-center randomized study reported that 8 weeks of IMT significantly improved the activity component of the St. George’s Respiratory Questionnaire (SGRQ) ^(13)^, 6MWD, and modified Medical Research Council (mMRC) dyspnea scale in patients with interstitial lung disease ^(14)^. Another randomized trial showed that training with the POWERbreathe Medic^Ⓡ^, a threshold IMT device, improved inspiratory muscle strength, 6MWD, and mMRC scores in patients with IPF ^(15)^. This study employs an improved version of this device, the POWERbreathe Medic Plus^Ⓡ^, which allows for easier load adjustment and safer use in patients with lower inspiratory pressures.

However, there remains no multicenter randomized controlled trial evaluating IMT specifically in IPF, and the long-term effects of IMT are unknown. In particular, the impact of IMT on activity-related quality-of-life domains has not been elucidated.

Herein, we describe the design of a multicenter randomized controlled trial (the Breathe IPF trial) evaluating both short- (8-week) and long-term (56-week) effects of IMT—particularly its impact on SGRQ scores—in patients with IPF. This study is a multicenter, open-label, parallel-group, randomized controlled trial designed to evaluate the efficacy and safety of IMT in patients with IPF.

## Methods

### Endpoints

The primary endpoint of this study is the change in the activity domain score of the SGRQ (SGRQ-A) from baseline to Week 8 (±1 day). The SGRQ comprises three domains—symptoms, activity, and impact—each scored from 0 to 100, with lower values indicating better HRQOL. A previous study in patients with interstitial lung disease demonstrated that 8 weeks of device-based IMT resulted in an improvement of approximately 9.83 points in the SGRQ-A compared with a control group. Because SGRQ-A reflects physical activity–related HRQOL and similar benefits are anticipated in patients with IPF, this domain was selected as the primary endpoint. A previous study has demonstrated that IMT preferentially improves SGRQ-A ^(14)^. Moreover, improving patient-reported outcomes is becoming a central therapeutic goal in IPF management, complementing traditional physiological endpoints.

The main secondary endpoints include changes in pulmonary functions, 6MWD, mMRC score, Dyspnea-12 score, the total score and individual domain scores of the SGRQ, psychological status evaluated by the Hospital Anxiety and Depression Scale, and safety outcomes over 56 weeks.

### Eligibility criteria

#### Inclusion criteria

Participants will be eligible for inclusion if they meet all of the following conditions:

1. Diagnosed with IPF according to the international American Thoracic Society, European Respiratory Society, Japanese Respiratory Society, and Asociacion Latinoamericana de Torax guidelines ^(16)^.

2. Receiving regular outpatient care at one of the seven participating tertiary hospitals in Japan.

3. Age between 40 and 85 years at the time of enrollment.

4. Stable clinical condition without acute exacerbation or pneumonia at the time of enrollment.

5. mMRC dyspnea grades 1-3.

6. Capable of performing IMT as instructed.

7. Able to provide written informed consent.

### Exclusion criteria

Participants will be excluded if any of the following conditions apply:

1. History of acute exacerbation of IPF or pneumonia within 3 months prior to enrollment.

2. History of pneumothorax or mediastinal emphysema.

3. Frequent exacerbations of asthma.

4. Ear injury, such as tympanic membrane perforation.

5. Abdominal hernia.

6. Pulmonary hypertension with an estimated pulmonary artery pressure of ≥45 mmHg on echocardiography.

7. Large pulmonary cysts detected on chest radiography.

8. History of bone fracture due to osteoporosis.

9. Abdominal surgery within 2 months prior to enrollment.

10. Receiving oxygen therapy at rest.

11. Unable to perform the 6-minute walk test (6MWT) or other required assessments due to severe mobility limitation (e.g., wheelchair use).

12. Uncontrolled malignant disease that could affect disease stability.

13. Aortic aneurysm.

14. Any other condition that, in the opinion of the investigator, makes participation inappropriate or unsafe.

### Study design

This study is designed to evaluate the efficacy and safety of IMT using the POWERbreathe Medic Plus (POWERbreathe International Ltd., Southam, Warwickshire, England, UK) in patients with IPF. Participants will be randomly assigned in a 1:1 ratio to either the IMT group or the control group receiving usual care without IMT using a centralized allocation system implemented through an electronic data capture system (TXP Medical Co., Ltd., Tokyo, Japan). Randomization will be performed using a stratified block randomization method with dynamic allocation. Stratification and adjustment factors will include age (cutoff: 75 years) and forced vital capacity (FVC; cutoff: 75%) of the predicted value.

Whenever feasible, outcome assessments, including the SGRQ and the 6MWT, will be conducted by assessors blinded to treatment allocation (assessor-blinded approach). The 6MWT will be performed according to a standardized protocol by trained personnel to ensure consistency across study sites. The SGRQ will be self-administered with minimal staff involvement to reduce the risk of influencing patient responses.

To minimize performance and expectation bias, standard care, follow-up schedules, lifestyle guidance, and pulmonary rehabilitation will be standardized across both groups. In addition, instructions for IMT will be provided in accordance with a predefined manual to ensure consistency and to avoid inducing excessive expectations.

A sham IMT intervention will not be implemented for ethical and methodological reasons, as even minimal loading can induce physiological effects and may not effectively eliminate placebo-related bias or potential risks.

The intervention period is 8 weeks, followed by a long-term follow-up of 56 weeks to assess sustained effects and safety.

The study protocol follows the Standard Protocol Items: Recommendations for Interventional Trials (SPIRIT) guidelines ^(17)^. The enrollment period for this study will be from April 22, 2025, to June 30, 2026, and the trial is scheduled to be completed on July 31, 2027. A schematic diagram of the study design is shown in Figure 1.

**Figure 1.**
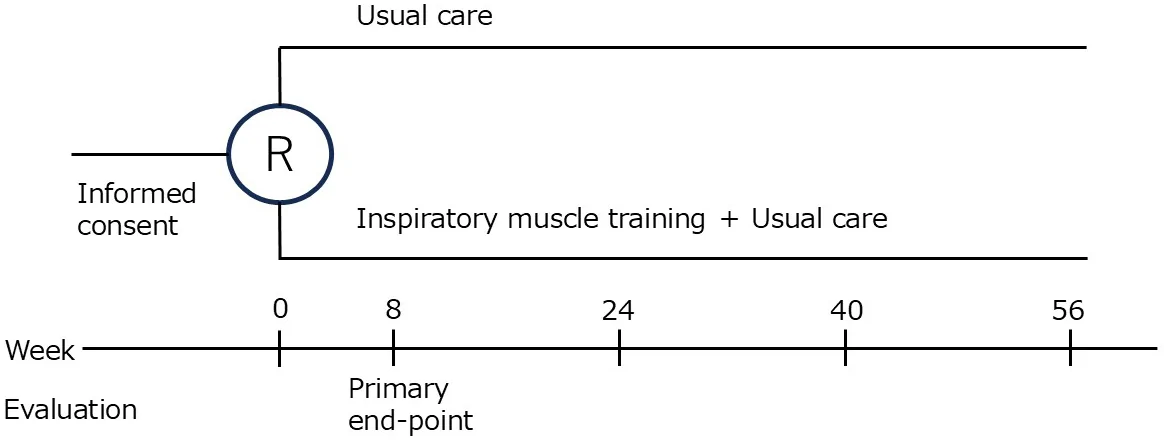
Study overview. After written consent is obtained, patients undergo baseline evaluation. After the randomization (Week 0), the control group will receive usual outpatient care (0-56 weeks). The inspiratory muscle training group will start inspiratory muscle training using the POWERbreathe Medic Plus and receive usual outpatient care (0-56 weeks).

### Participant recruitment

Participants will be recruited from the outpatient departments of the following seven tertiary hospitals in Japan: Nagoya City University Hospital, Nagoya City University Midori Municipal Hospital, Nagoya City University East Medical Center, Fujita Health University Hospital, Kyoto University Hospital, Tokyo Medical University Hospital, and Shiga University of Medical Science Hospital.

Patients who meet the eligibility criteria will receive detailed oral and written explanations regarding the study objectives, procedures, potential benefits, and possible risks. After being given sufficient time to consider participation, patients who agree to take part will provide written informed consent before undergoing any study-related assessments. Recruitment will be conducted concurrently at all seven centers.

### Intervention

#### Preintervention period (2-8 weeks before Day 0)

During the preobservation period, all eligible participants will undergo baseline assessments, including echocardiography, electrocardiography, chest radiography, and routine blood examinations, to confirm eligibility and document preintervention status.

Participants will also receive an educational booklet titled “Respiratory Rehabilitation for Living Well with Interstitial Lung Disease” (supervised by Prof. Rei Kozu, Nagasaki University; Boehringer Ingelheim Japan), which encourages voluntary physical activities such as knee extension, tiptoe standing, squatting, and walking to promote general conditioning before the initiation of IMT. The booklet consists of 25 pages.

#### Intervention period (Day 0-Week 56)

##### Common procedures for both groups

Maximal inspiratory mouth pressure (PImax) will be measured on Day 0 and at Weeks 8, 24, 40, and 56 using a handheld respiratory muscle strength meter (IOP-01; Kobata Keiki Seisakusho Co., Ltd., Osaka, Japan).

Patients in both groups will be encouraged to perform home-based rehabilitation throughout the study period, up to 56 weeks, using the aforementioned educational booklet titled “Pulmonary Rehabilitation for Living Better with Interstitial Lung Disease.” Patients who had been receiving outpatient or day-care rehabilitation before enrollment will be allowed to continue their existing rehabilitation programs.

Both groups will receive usual care, including guidance for home-based exercise using the rehabilitation booklet. This booklet-based program is intended to reflect routine clinical practice in Japan, where home-based pulmonary rehabilitation is commonly encouraged through educational materials. Adherence to the booklet-based exercise program will not be formally or quantitatively monitored, and no adherence-based subgroup analyses are planned. Although adherence is not formally monitored, participants will be routinely encouraged to follow the booklet-based program during study visits. Basic physical activity guidance will be provided equally to both groups; the only difference between the groups will be the addition of IMT in the intervention group.

Pharmacological treatments, including antifibrotic therapy and steroid treatment, will be administered at the discretion of the treating physicians and are independent of this study.

### IMT group

Participants assigned to the IMT group will perform home-based training using a threshold loading device (POWERbreathe Medic Plus; Figure 2) for a total of 56 weeks.

**Figure 2.**
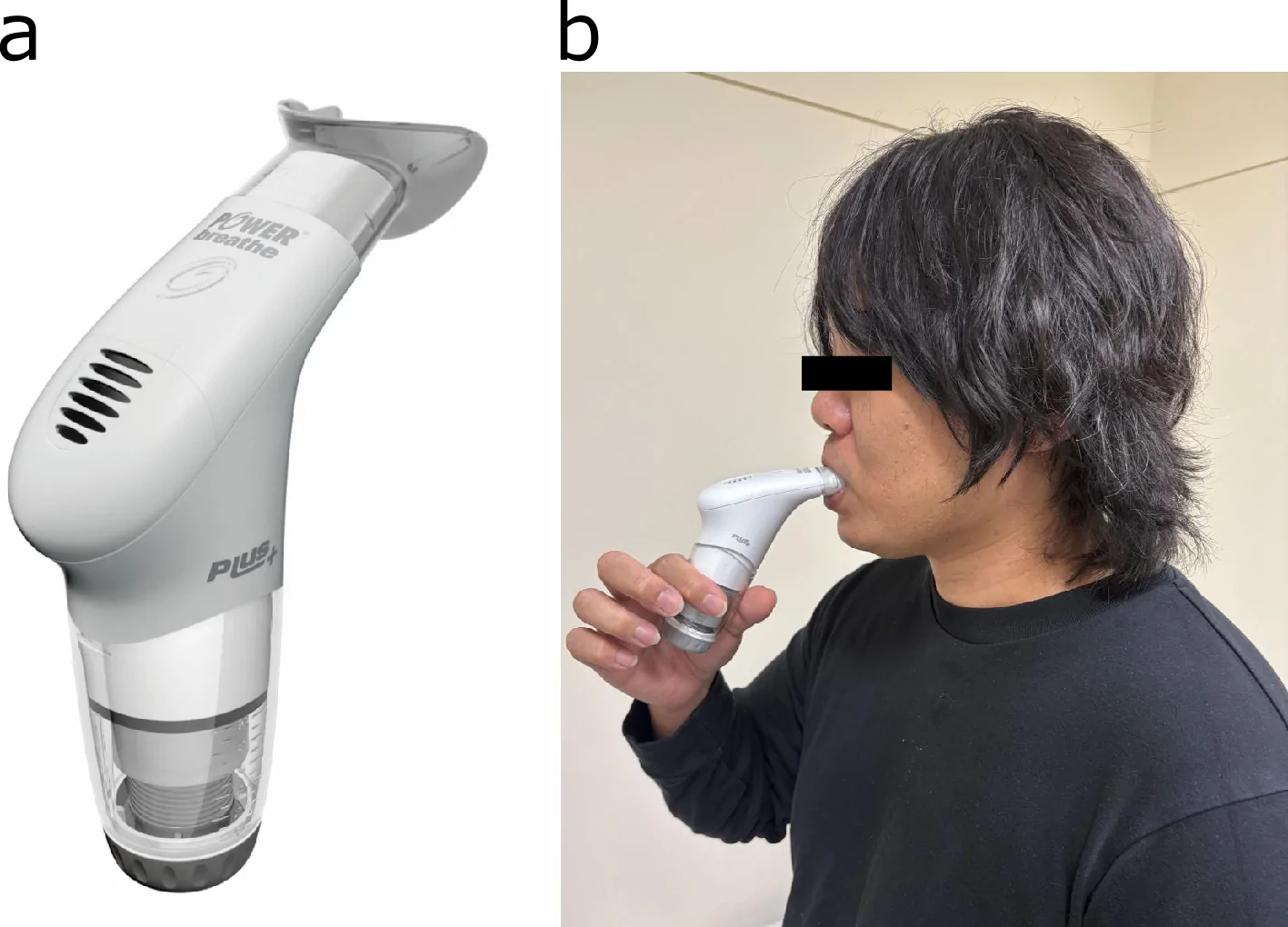
Threshold loading device for inspiratory muscle training. (a) Threshold loading device for inspiratory muscle training (POWERbreathe Medic Plus). (b) Inspiratory muscle training being performed.

Each session will consist of 30 breaths, performed twice daily. The initial training load will be set at approximately 30% of PImax for the first 2 weeks, increased to 40% from Week 3, 50% from Week 5, and 60% from Week 7 onward. For participants with PImax ≥118 cmH_2_O, the maximal resistance (Level 9) will be used.

PImax will be remeasured at Weeks 8, 24, and 40 to confirm the training effect and to adjust the load as needed. Participants will continue IMT at approximately 60% of the updated PImax throughout the 56-week period. Training adherence will be monitored using participants’ training records reviewed at scheduled visits.

Adherence to IMT will be assessed primarily using patient self-reported training records. The IMT device used in this study does not have a built-in function for objective adherence monitoring; therefore, device-based monitoring, electronic training logs, and telemonitoring will not be implemented.

To support adherence, participants will be followed up at each scheduled outpatient visit, during which training status will be reviewed and reinforced by study staff according to standardized procedures. Self-reported training records will also be checked by healthcare providers.

The study flow is summarized in Figure 1. After the randomization (Week 0), the control group will receive usual outpatient care (0-56 weeks). The IMT group will start IMT using the POWERbreathe Medic Plus and receive usual outpatient care (0-56 weeks).

#### Assessment schedule and follow-up

Assessments will be conducted at 8, 24, 40, and 56 weeks during the 56-week intervention period, and details of the primary and secondary outcome measures, including their definitions and timing, are described in the Data collection subsection. After completion of the 56-week intervention, all participants will be followed up until Week 64 (±2 weeks) for safety monitoring, and those in the IMT group who wish to continue training after 56 weeks may do so voluntarily at approximately 60% of PImax.

### Data collection

All outcomes will be assessed at baseline and at 8, 24, 40, and 56 weeks after the start of the intervention, with a safety follow-up at 64 weeks (±2 weeks). All data will be entered into the electronic data capture system by trained investigators, and the overall assessment schedule is summarized in Table 1.

**Table 1.** Schedule of Assessments.

Items	Times					
	Baseline	8 weeks	24 weeks	40 weeks	56 weeks
Patient characteristics	●				
SpO_2_	●	●	●	●	●
Chest x-ray	●	●	●	●	●
Spirometry	●	●	●	●	●
DLco	●	●			●
6MWT	●	●			●
PImax	●	●	●	●	●
SGRQ	●	●	●	●	●
mMRC	●	●	●	●	●
Dyspnea-12	●	●	●	●	●
HADS	●	●	●	●	●
Adverse events	●	●	●	●	●

6MWT: 6-minute walk test; DLco: diffusing capacity of the lung for carbon monoxide; HADS: Hospital Anxiety and Depression Scale; mMRC: modified Medical Research Council; PImax: maximal inspiratory mouth pressure; SGRQ: St. George’s Respiratory Questionnaire; SpO_2_: peripheral oxygen saturation.

Pulmonary function tests, including diffusing capacity of the lung for carbon monoxide (DLco), will be performed at baseline, 8 weeks, and 56 weeks to assess disease activity. These assessments will not be conducted at 24 and 40 weeks to minimize patient burden.

At each scheduled visit, investigators will record the use of home oxygen therapy and monitor adverse events (AEs).

AEs potentially related to IMT, including dizziness, syncope, respiratory discomfort, and barotrauma, will be prospectively monitored throughout the study period. All AEs will be systematically collected and recorded, and each event will be classified according to its severity and assessed for its causal relationship with the intervention based on predefined criteria.

Serious AEs will be promptly reported to the principal investigator by the responsible physician at each site. Appropriate medical management will be provided, and continuation of the intervention will be evaluated on a case-by-case basis.

Given the low-risk and noninvasive nature of the intervention, an independent data safety monitoring board will not be established. Instead, safety data will be regularly reviewed by the principal investigator and the study team to ensure ongoing safety oversight.

The intervention will be discontinued for individual participants if safety concerns arise based on clinical judgment. The study may be terminated if significant safety concerns emerge or if continuation is deemed inappropriate by the principal investigator.

### Statistical analysis

#### Sample size

The sample size was calculated based on the expected between-group difference in the SGRQ-A, using estimates from previous studies in patients with interstitial lung disease ^(14)^. The reported mean difference in SGRQ-A was 9.83, with a standard deviation of 10.38. Based on a two-sided significance level of 0.05 and a statistical power of 0.90, a total sample size of 49 participants was estimated to be sufficient to detect a clinically meaningful difference between the groups.

Considering a dropout rate of approximately 15%-20%, based on previous studies in patients with IPF and the relatively long follow-up period of 1 year ^(18)^, the target sample size was set at 60 participants (30 per group) to ensure adequate statistical power.

In addition, the estimated sample size required to detect a difference in the 6MWD, a secondary outcome, was 45 participants, which is lower than the planned sample size.

#### Outcome analysis

The analyses for efficacy will be performed in the full analysis set comprising all randomized patients who had undergone baseline assessment and at least one evaluation point following randomization. Sensitivity analyses will be performed using a per-protocol set. The per-protocol set will be defined as all patients who met the prespecified criteria. Data handling will be defined for each endpoint. A mixed-effects model for repeated measures will be applied to the comparison of the change in SGRQ-A from baseline between treatment groups with a significance level of 5% (two-sided). The least squares mean and 95% confidence interval will be calculated using a linear mixed-effects model including treatment group, SGRQ-A at baseline, evaluation time point, and an interaction term of the treatment group and evaluation time point as fixed effects. Missing data will not be imputed. All analyses will be performed on an intention-to-treat basis including all randomized participants with at least one postbaseline assessment. Other secondary endpoints, dyspnea (Dyspnea-12 and mMRC scores), total score and individual domain scores of SGRQ, %FVC, %DLco, and 6MWD will be evaluated in the same manner. Demographic and clinical characteristics will be analyzed using t tests and Fisher’s exact tests, respectively. AEs will be summarized and compared using Fisher’s exact test. For all statistical analyses, statistical significance will be set at 0.05. Interim analyses will be planned to be conducted when 50 patients have been enrolled and again 24 weeks thereafter. All analyses will be performed using SPSS version 28.0 (IBM Corp., Armonk, NY, USA) and JMP (SAS Institute Inc., Cary, NC, USA).

## Discussion

IPF imposes profound functional limitations and a substantial symptom burden despite current pharmacological therapy ^(4), (6)^. Against this background, IMT represents a particularly relevant therapeutic strategy because it directly targets respiratory muscle dysfunction while being feasible for integration into daily life. Approaches that support day-to-day functioning and help patients remain active are therefore essential complements to disease-modifying treatment. From this perspective, IMT may contribute to preserving respiratory performance and mitigating activity-related declines in health status over time.

Evidence from pulmonary rehabilitation studies including IMT or breathing exercises suggests that such interventions can improve exercise capacity, dyspnea, and HRQOL in patients with IPF and interstitial lung disease ^(14), (15)^. Aerobic training combined with breathing exercises or IMT has been associated with gains in 6MWD, reduced dyspnea, and improved HRQOL compared with control conditions in systematic reviews and randomized trials ^(19)^.

One of the notable strengths of this study is its emphasis on patient-centered outcomes, particularly the SGRQ-A. These measures reflect aspects of daily life that are highly important to patients with IPF; yet, they have been insufficiently addressed in conventional clinical trials that tend to focus primarily on objective endpoints such as pulmonary function tests and exercise capacity. If IMT favorably influences these patient-reported outcomes, it would suggest that IMT provides a practical, clinically meaningful intervention that extends beyond physiological improvement to support self-management. This perspective is consistent with existing literature emphasizing improvements in dyspnea and quality-of-life measures through comprehensive rehabilitation approaches that include respiratory muscle training ^(11), (14)^.

This pragmatic, multicenter trial investigates IMT as an adjunctive intervention under real-world clinical conditions. By implementing the intervention across multiple university hospitals, the study seeks to determine whether IMT confers additional benefits beyond standard pharmacological treatment and routine clinical care. Usual care in this trial consists of providing an educational booklet titled “Respiratory Rehabilitation for Living Well with Interstitial Lung Disease” (supervised by Prof. Rei Kozu, Nagasaki University), along with ongoing encouragement to engage in home-based rehabilitation throughout the study period. The booklet promotes self-directed physical activities—such as knee extension, tiptoe standing, squatting, and walking—to support general physical conditioning prior to the initiation of IMT. Participants who were already undergoing outpatient or day-care rehabilitation at the time of enrollment were permitted to continue their existing rehabilitation regimens.

Although assessed as a secondary endpoint, a key novel aspect of this study is the evaluation of both the long-term efficacy and safety of IMT over a 56-week period. To our knowledge, no previous studies in patients with IPF have examined the sustained effects of structured IMT, together with the assessment of potential AEs, over such an extended follow-up duration.

Several limitations warrant consideration. Due to its open-label design and the use of a patient-reported primary outcome, performance and expectation bias cannot be fully excluded. Although an assessor-blinded approach will be implemented whenever feasible, complete blinding is not possible. The sample size of this study is modest, which may limit statistical power and make meaningful subgroup analyses difficult. Although this is a multicenter study with potential variability across sites, the sample size was determined based on an expected effect size derived from prior studies and is considered sufficient to detect a clinically meaningful difference in the primary outcome. In addition, because the intervention will be delivered as home-based training, adherence may vary among participants and could influence treatment effects. Adherence is assessed based on self-reported training records, which may introduce reporting bias, particularly given the long duration of the intervention. Furthermore, the primary endpoint of this study, the SGRQ-A, is a subjective patient-reported outcome, and in theory, the use of a sham IMT control could reduce potential bias. However, a sham intervention will not be implemented because participants are expected to readily recognize it as such. Nonetheless, these features will reflect real-world implementation and are expected to provide valuable insights into feasibility and acceptability in routine clinical practice.

In summary, this trial aims to determine whether adding structured IMT to usual care improves activity-related health status in patients with IPF. The results are expected to clarify the role of simple, home-based respiratory training as a component of long-term disease management. Enrollment is ongoing, and the trial is designed to provide robust evidence regarding the long-term efficacy of IMT in IPF.

## Article Information

### Author Contributions

All authors contributed to the design of the “Breathe IPF trial” and the writing of this manuscript. Akihito Okada drafted the manuscript. Hirotsugu Ohkubo is responsible for the overall content as the guarantor. All authors have read and approved the final version of this manuscript.

### Conflicts of Interest

Tomohiro Handa is employed by a collaborative research laboratory funded by Teijin Pharma Co., Ltd., and has received research grants from FUJIFILM Corporation. The other authors have no conflicts of interest to declare.

### Ethical Considerations

This trial will be conducted in accordance with the Declaration of Helsinki and the Ethical Guidelines for Medical and Biological Research Involving Human Subjects in Japan, as well as Good Clinical Practice principles. Written informed consent will be obtained from all participants prior to enrollment. This study protocol has been approved by the Institutional Review Board of Nagoya City University Hospital (approval number: 2024B002).

### Protocol Registration

The protocol was registered on the Japan Registry of Clinical Trials website (protocol ID: jRCT1042250003) on April 22, 2025.
